# *Drosophila* wing imaginal discs respond to mechanical injury via slow InsP_3_R-mediated intercellular calcium waves

**DOI:** 10.1038/ncomms12450

**Published:** 2016-08-09

**Authors:** Simon Restrepo, Konrad Basler

**Affiliations:** 1 Institute of Molecular Life Sciences, University of Zurich, Winterthurerstrasse 190, Zurich CH-8057, Switzerland

## Abstract

Calcium signalling is a highly versatile cellular communication system that modulates basic functions such as cell contractility, essential steps of animal development such as fertilization and higher-order processes such as memory. We probed the function of calcium signalling in *Drosophila* wing imaginal discs through a combination of *ex vivo* and *in vivo* imaging and genetic analysis. Here we discover that wing discs display slow, long-range intercellular calcium waves (ICWs) when mechanically stressed *in vivo* or cultured *ex vivo*. These slow imaginal disc intercellular calcium waves (SIDICs) are mediated by the inositol-3-phosphate receptor, the endoplasmic reticulum (ER) calcium pump SERCA and the key gap junction component Inx2. The knockdown of genes required for SIDIC formation and propagation negatively affects wing disc recovery after mechanical injury. Our results reveal a role for ICWs in wing disc homoeostasis and highlight the utility of the wing disc as a model for calcium signalling studies.

## Abstract (web summary)

It is unclear what role calcium signalling plays in the *Drosophila* wing disc. Here, the authors show that on mechanical stress, slow, long-range intercellular calcium waves are initiated *in vivo* and *ex vivo*, mediated by the inositol-3-phosphate receptor, the calcium pump SERCA and gap junction component Inx2.

## Graphical abstract

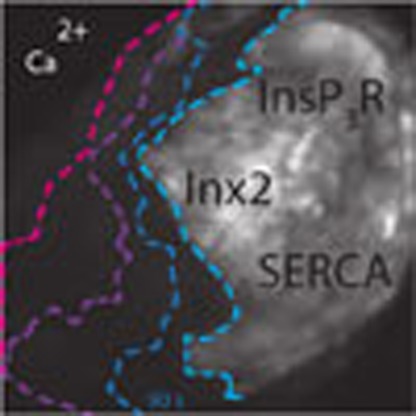

Calcium (Ca^2+^) is a ubiquitous and highly versatile signal that modulates basic cellular functions, such as contractility and secretion, essential steps of animal development such as fertilization and proliferation, and higher-order processes such as learning and memory^1^,^2^,^3^. Intercellular calcium waves (ICWs) constitute an interesting aspect of Ca^2+^ signalling currently been untangled^4^,^5^.

ICWs have been observed in a variety of systems: *in vivo* and *ex vivo*^4^,^5^,^6^,^7^. In zebrafish^8^,^9^ and *Xenopus*^10^,^11^,^12^,^13^, ICWs occur during important developmental milestones such as gastrulation^2^. However, the functional relevance of ICWs often remains open^1^,^2^.

ICWs have been reported to occur during specific stages of *Drosophila* embryonic development^14^. Recently, calcium flashes—a fast type of ICWs—have been shown to regulate inflammation and the response to injury in the fly embryo^15^. Further, it was also recently found that calcium signalling might regulate coordinated waves of actomyosin flow and cell constriction on wounding of the *Drosophila* pupal notum^16^.

Yet, calcium signalling has seldom been explored in wing imaginal discs, arguably one of the better-studied model organs. To redress this we decided to use our recent development of a system for *ex vivo* culturing of imaginal discs^17^ and improvements in ultrasensitive genetically encoded calcium indicators, the GCamp6 reporters^18^.

Through *ex vivo* live imaging of wing discs expressing GCamP6s^18^, we uncover a slow type of ICW that we named slow imaginal disc ICW (SIDIC). Next, we characterize the molecular mechanism underlying the SIDICs through a combination of quantitative live imaging of ICWs and gene knockdown analysis. We find that the SIDICs are a cell-to-cell chain of intracellular Ca^2+^ release from the endoplasmic reticulum (ER) Ca^2+^ stores downstream of the inositol-3-phosphate receptor (InsP_3_R) that requires gap junctions for intercellular propagation. Further, we find that, *in vivo*, the SIDICs constitute a response to mechanical stress. Finally, we explore whether the SIDICs might play a role as an injury response. We find that the knockdown of the InsP_3_R and the key gap junction component innexin2 (Inx2) affect the wound healing of wing discs negatively. Our results highlight a role for calcium signalling in the form of slow, long-range InsP_3_R-mediated Ca^2+^ waves in the homeostasis of wing imaginal discs.

## Results

### *Ex vivo*-cultured imaginal wing discs display ICWs

**Figure 1:**
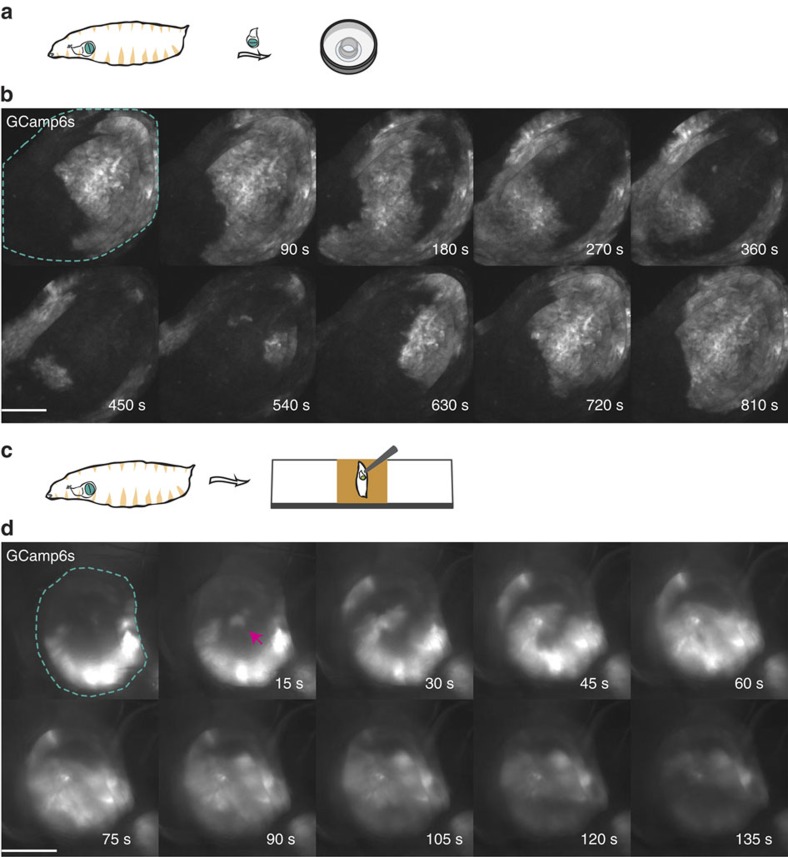
Observation of intercellular calcium waves in imaginal wing discs *ex vivo* and *in vivo*. (**a**) *Ex vivo* imaging setup. Dissected wing discs are cultured in WM1 inside a modified cell culture insert on an imaging disc and imaged with an inverted spinning-disc confocal microscope. Fly strains: *nub>GCamp6s X yw.* (**b**) Example of a SIDIC wave *ex vivo*. This wave propagated approximately along the antero-posterior axis of the wing disc and recruited all of the cells of the wing pouch. It is noteworthy how the wave starts to recur after 540 s. Scale bar, 100 μm. Fly strains: *nub>GCamp6s X yw.* (**c**) *In* vivo imaging setup (inverted). Larvae were glued to a microscope slide, dorsally, with double-sided adhesive tape. We employed an inverted widefield fluorescence microscope. Fly strains: *nub>GCamp6s X yw.* (**d**) Observation of a SIDIC wave *in vivo.* The magenta arrowhead indicates the point were the SIDIC originates. Scale bar, 100 μm. Fly strains: *nub>GCamp6s X yw.*

To study calcium signalling activity in imaginal wing discs, we employed a previously developed *ex vivo* culturing setup that is amenable to high-resolution live imaging and supports normal levels of proliferation for up to 12 h (ref. 17). We employed a genetically encoded calcium indicator, UAS-GCamp6s, under the control of a wing pouch driver (nubbin-Gal4). We refer to this fly strain as *nub>GCamP6s*. Strikingly, we observed large SIDICs propagating across the wing imaginal disc explants (Fig. 1a,b and Supplementary Movie 1).

To better understand this phenomenon, we characterized the properties of the SIDICs (Supplementary Fig. 1A–J). The SIDIC wavefront is slow at ∼0.4 μms^−1^ (Supplementary Fig. 1A,B: 0.41±0.17, *n*=10 discs). During each wave, the recruited imaginal disc cells mobilize intracellular calcium for nearly 6 min (Supplementary Fig. 1C–E: 6.18±1.97, *n*=10 discs). *Ex vivo*, 75% (*n*=41/55 discs) of SIDICs propagate throughout the whole pouch with a magnitude (area covered by wave/pouch area) close to 1 (Supplementary Fig. 1F–G: 0.99±0.01, *n*=10 discs). In 13% of the discs the SIDICs were of smaller magnitude with only a fraction of the cells of the wing pouch being recruited (0.43±0.30, *n*=9). Finally, an additional 12% did not display SIDIC activity. *Ex vivo*, the SIDICs recur with a relatively constant period (Supplementary Fig. 1H–J: 13.9 min±3.4, *n*=22 discs). In addition, per disc the spatio-temporal pattern of propagation is generally repeated (Fig. 1b and Supplementary Movie 1). Both the duration of the recurrence period and the spatio-temporal pattern of wave propagation vary more among discs than per disc[Fig f2].

### The SIDICs are induced by mechanical stress

ICWs can be induced by mechanical stimulation and mechanical wounding *in vitro* in airway epithelial cells^19^ and human urothelial cell monolayers^20^. Further, during our first trials at *in vivo* imaging of SIDICs we had found that SIDICs could only be observed if the larvae were squeezed in between a coverslip and a glass slide in a setup that resulted in mechanical stress for the larva (Supplementary Fig. 2). We also noted that sometimes the SIDICs seemed to occur after strong larval movements, again pointing towards mechanical stress as a trigger.

We therefore tested whether mechanical stress could trigger a SIDIC wave *in vivo.* We developed a setup in which we glued larvae, imaginal discs facing down, on a microscope slide with double sided adhesive tape (Fig. 1c). Next, we used blunt forceps to punch the imaginal discs through the larval cuticle, but crucially, without piercing the cuticle. We found that this manipulation induced two different calcium responses. First, there is a direct strong and localized increase in intracellular calcium levels (dashed lines in Fig. 3). Further, in 63% of perturbed discs (*n*=10/16) we observed a SIDIC wave appearing during the next 30 min (Fig. 1d and Supplementary Movie 2). In contrast, we only observed SIDICs in 9.8% (*n*=4/41) of unperturbed discs. The presence of SIDICs in unperturbed discs could indicate a different potential source of stimulation for the SIDICs (for example, mechanical stimulation induced by larval movements during foraging, before imaging) or that the discs were sometimes involuntarily mechanically stressed, while the larvae were glued onto the adhesive tape. The delay between the mechanical stimulation and the SIDICs suggests that these waves constitute a later response to injury and are in this respect different from the fast calcium flashes described in the embryo^15^ or notum^16^.

The SIDICs induced by mechanical stress happen on a similar timeframe as those observed *ex vivo* and require several minutes to spread through the wing disc (Fig. 1d). The SIDICs are difficult to characterize *in vivo* due to larval movements. However, we found the following approximations: the wavefront speed is of 0.76±0.42 μms^−1^ (*n*=8); the duration of intracellular calcium mobilization seems shorter at 3.7±1.2 min (*n*=8); the magnitude is generally smaller and more variable (0.36±0.28, *n*=8); and there seems to be no constant recurring period, although we did observe recurring waves (*n*=5/16) (Supplementary Movie 3). The disparities in wave magnitude could reflect differences in the type and magnitude of stimuli inducing the waves. *In vivo* the trigger is a transient mechanical stress, whereas *ex vivo* the stimuli are linked to the dissection and culturing conditions, and are likely to be stronger and constantly present.

### The SIDICs mobilize intracellular Ca^2+^ via InsP_3_R and SERCA

The magnitude of the SIDICs and the constant intensity of GCamP6s fluorescence during propagation hint to a calcium-induced calcium release mechanism of propagation. Calcium-induced calcium release can be induced by the InsP_3_R and the ryanodine receptor (RYR), sometimes exclusively and sometimes in combination^1^,^2^,^3^,^5^. Previous analyses of RYR expression and function concluded that RYR was only important for muscle function in *Drosophila*^21^,^22^,^23^,^24^,^25^. We therefore focused on InsP_3_R.

**Figure 2 f2:**
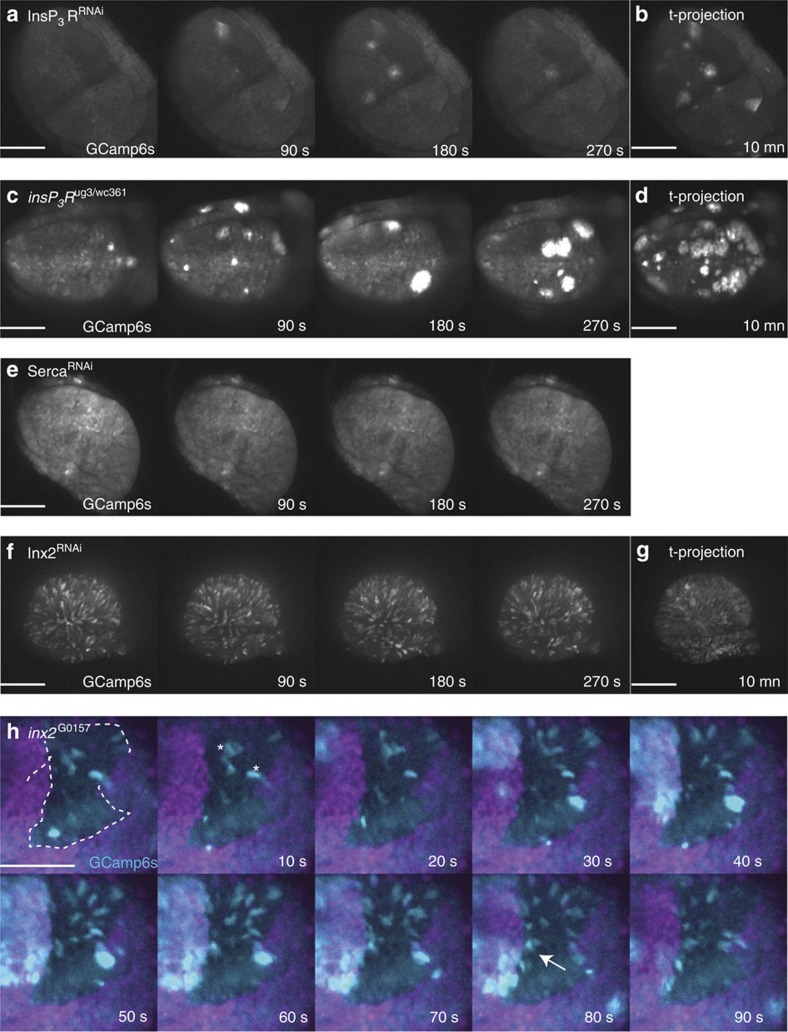
Characterization of key genes required for SIDIC wave propagation *ex vivo*. (**a**) InsP3R knockdown (InsP_3_R^RNAi^) prevents the formation of the SIDICs. The presence of groups of cells undergoing intracellular calcium transients, which we refer to as calcium bursts, are noteworthy. Scale bar, 100 μm. Fly strains: *nub>GCamP6s X UAS-InsP*_*3*_*R*^*RNAi*^ (NIG 1063-R1). (**b**) Ten minutes time projection of GCamp6s fluorescence for InsP_3_R^RNAi^. Scale bar, 100 μm. Fly strains: *nub>GCamP6s X UAS-InsP*_*3*_*R*^*RNAi*^ (NIG 1063-R1). (**c**) *Trans*-heterozygous hypomorphs for *InsP*_*3*_*R* (^*ug3/wc361*^) InsP_3_R^RNAi^. The calcium transient activity is more intense, probably revealing higher residual InsP_3_R activity. Scale bar, 100 μm. Fly strains: *InsP*_*3*_*R*^*ug3*^ *X InsP*_*3*_*R*^*wc361*^. (**d**) Ten minutes time projection of GCamp6s fluorescence for *InsP*_*3*_*R*^ug3/wc361^. Scale bar, 100 μm. Fly strains: *InsP*_*3*_*R*^*ug3*^ *X InsP*_*3*_*R*^*wc361*^. (**e**) RNAi-mediated knockdown of the ER calcium pump SERCA (SERCA^RNAi^) prevents the formation of the SIDICs consistent with the requirement of intracellular Ca^2+^ stores for the SIDICs. Scale bar, 100 μm. Fly strains: *nub>GCamP6s X UAS-SERCA*^*RNAi*^ (BL 25928). Similar results were obtained with BL 44581. (**f**) An interfering RNA against the gap junction component Inx2 (Inx2^RNAi^) prevents the formation of the SIDICs. Scale bar, 100 μm. Fly strains: *nub>GCamP6s X UAS-Inx2*^*RNAi*^ (BL 29306). Similar results were obtained with UAS-wizInx2. (**g**) Ten minutes time projection of GCamp6s fluorescence for Inx2^RNAi^. Scale bar, 100 μm. Fly strains: *nub>GCamP6s X UAS-Inx2*^*RNAi*^ (BL 29306). (**h**) A SIDIC wave stopping at the boundary of an Inx2 mutant clone generated by somatic recombination (*inx2*^*G0157* −*/*−^). The wave ends at the interior boundary of the clone (white arrow indicates increased GCamP6s fluorescence at the inner boundary of the clone). The presence, inside the clone, of the same calcium ‘sparkles' observed in Inx2^RNAi^ discs are noteworthy. Scale bar, 50 μm. Fly strains: *w, inx2*^*G0157*^*, FRT19A/FM7c* X *ubi>mRFPNLS,w,hsflp,FRT19; nubbin-Gal4,GCamP6s; MKRS/Tm6b.*

The knockdown of the InsP_3_R abolished the SIDICs *ex vivo* (Fig. 2a,b and Supplementary Movie 4). During InsP_3_R knockdown, in place of SIDICs we observed spontaneous occurrences of intracellular calcium release dispersed discretely throughout the wing pouch (Supplementary Fig. 3A and Supplementary Movie 4). These ‘bursts' initiate in a small number of neighbouring cells and can propagate for one to two cell diameters (Supplementary Fig. 3B). The duration of calcium mobilization during a burst is shorter than during a SIDIC wave (44.3±18.5 s, *n*=23 versus 6.18 min, 1±1.97). However, the speed at which these bursts propagate is comparable to that of the SIDICs wavefront (0.51±0.16 μms^−1^, *n*=18). In some cases, the bursts can be quite large, involving up to ∼50 cells. However, these bursts never lead to the propagation of a SIDIC wave. We confirmed these RNA interference (RNAi) results by generating a *trans*-heterozygous hypomorph combinations of *InsP*_*3*_*R* alleles (*ug3/wc361*)^26^. The *trans*-heterozygous situation mimicked the RNAi experiments but the calcium activity seemed higher (Fig. 2c,d). This could indicate that the calcium bursts are a result of residual InsP_3_R activity present in both the knockdown and hypomorph situations.

Taken together, these experiments suggest that the calcium bursts represent SIDICs that failed to propagate due to insufficient InsP_3_R activity. This could indicate that intracellular Ca^2+^ has to be mobilized for a sufficiently long duration before the SIDICs can propagate from one cell to the next. However, these bursts could also point to other necessary components for SIDIC activity that remain to be uncovered.

On activation, the InsP_3_R mobilizes Ca^2+^ from the intracellular Ca^2+^ stores of the ER. The ER calcium pump SERCA is required for the maintenance of the ER Ca^2+^ stores^1^,^27^. Consistent with the notion that the mobilization of intracellular Ca^2+^ from the ER is necessary for the propagation of the SIDICs, SERCA knockdown abolished the SIDICs (Fig. 2e and Supplementary Movie 5).

### The SIDICs require Inx2 for propagation

ICWs propagate via two major mechanisms: gap junctions and paracrine signalling with an extracellular messenger^5^. The ligand of the InsP_3_R, IP3 diffuses through gap junctions^5^. Hence, we tested whether gap junctions were required for SIDIC propagation by knocking down the key gap junction component Inx2 (ref. 28). Discs with impaired gap junction communication did not display SIDICs (Fig. 2f and Supplementary Movie 6). Time-lapse analysis of Inx2 knockdown experiments revealed a striking, sparkle-like pattern of intracellular Ca^2+^ release comprising either a single cell or small groups of adjacent cells (Supplementary Fig. 4A). These calcium pulses occur seemingly randomly throughout the wing disc epithelium and could sometimes be seen to propagate across neighbouring cells for short distances (Supplementary Fig. 4A). These results could support a role for gap junctions in SIDIC wave propagation. However, to rule out that loss of optimal gap junction function could be leading to general effects, for example, on calcium homeostasis, we performed *ex vivo* time-lapse analysis of wing discs with *inx2* mutant clones (*inx2*^*G0157*−*/*−^). Time-lapse analysis of *inx2* mutant clones generated by flippase recognition target (FRT)-mediated somatic recombination revealed that the SIDICs could not fully propagate across the clones and either stop at the clone border or propagate for a few cell diameters (Fig. 2h and Supplementary Movie 7). In addition, the same sparkles that were observed in the knockdown experiments could be observed inside the mutant clones (Fig. 2h and Supplementary Movie 7). We conclude that the SIDICs cannot fully propagate through cells that have impaired gap junction function. Here we identified InsP_3_R, SERCA and the key gap junction component Inx2 as SIDIC components. Future work will be necessary to uncover additional SIDIC modulators and fully reveal the propagation mechanism.

### RNAi targeting key SIDIC prevents SIDIC formation *in vivo*

**Figure 3:**
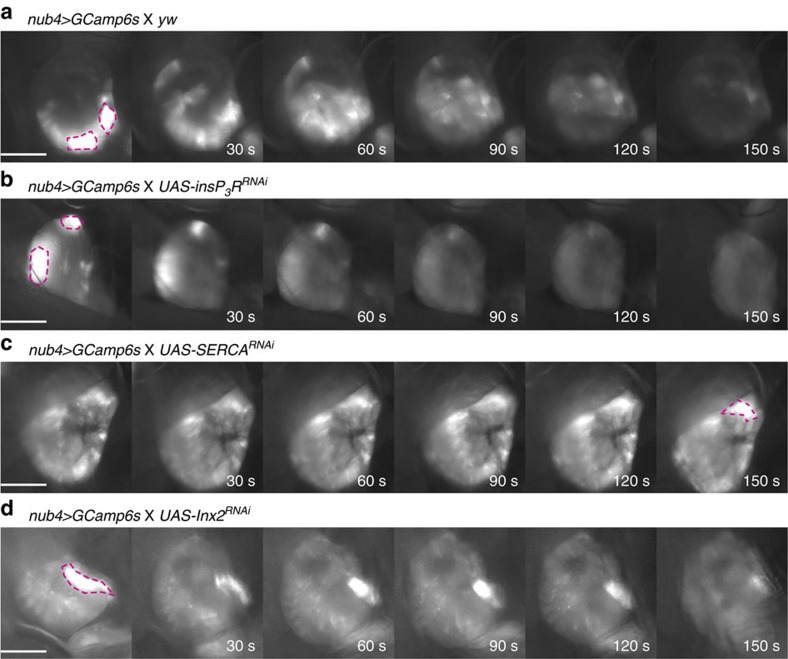
The SIDICs components identified *ex vivo* are required *in vivo*. (**a**) Control wing discs of the nub4>GCamp6s genotype displaying a SIDIC after mechanical induction (same time lapse as Fig. 1d). SIDICs were observed in 63% of mechanical stimulation experiments during a 30 min time window (10/16). Scale bar, 100 μm. Fly strains: *nub>GCamp6s X yw.* (**b**) InsP_3_R knockdown prevents the formation of SIDICs, *in vivo* (0/14). Scale bar, 100 μm. Fly strains: *nub>GCamP6s X UAS-InsP*_*3*_*R*^*RNAi*^ (NIG 1063-R1). (**c**) SERCA^RNAi^ prevents the formation of SIDICs *in vivo* (0/7). Scale bar, 100 μm. Fly strains: *nub>GCamP6s X UAS-SERCA*^*RNAi*^ (BL 25928). (**d**) Inx2^RNAi^ prevents the formation of SIDICs, *in vivo* (0/9). Scale bar, 100 μm. Fly strains: *nub>GCamP6s X UAS-Inx2*^*RNAi*^ (BL 29306). (**a**,**b**,**c**,**d**) Dashed lines highlight areas of immediate calcium release. These areas do not expand as a SIDIC wave.

Having identified InsP_3_R, SERCA and Inx2 as SIDICs components *ex vivo*, we asked whether the knockdown of these genes would prevent the formation of SIDICs *in vivo*. We stimulated SIDIC formation by striking the wing discs through the cuticle and monitoring them for a 30-min time window. As mentioned previously, striking wing discs *in vivo* triggers two calcium-mediated reactions: a localized initial increase in intracellular calcium that does not propagate as a slow wave (dashed lines; Fig. 3) and later a SIDIC wave (Fig. 1d). All genotypes displayed the first type of reaction (in SERCA^RNAi^ discs it seems delayed and of lower amplitude; Fig. 3c). However, although in control larvae 63% of stroked discs displayed a SIDIC wave during the time window of observation, we did not observe any SIDICs for InsP3R^RNAi^ (*n*=0/14), SERCA^RNAi^ (*n*=0/7) or Inx2^RNAi^ (*n*=0/9) (Fig. 3a–d). Interestingly, the Ca^2+^ ‘sparkles' that we observed *ex vivo* in Inx2^RNAi^ wing discs could only be observed *in vivo* after mechanical stress (Supplementary Fig. 4B), revealing that these constitute a reaction to injury and culture, and not a basal change in gap junction function. The *in vivo* results confirm that the SIDICs observed *ex vivo* are a close proxy and adequate model of the *in vivo* phenomenon. Given the difficulty of accurately characterizing the SIDICs *in vivo*, an *ex vivo* model will be a useful tool to further identify required components and to continue dissecting the propagation mechanism of the SIDICs.

### Knockdown of InsP_3_R and Inx2 impairs recovery after injury

We next asked what might be the function of the SIDICs during wing imaginal disc development. Previous studies have spotlighted the role of calcium signalling in wound healing and regeneration^15^,^16^,^29^,^30^,^31^,^32^. Further, our *in vivo* experiments had revealed that the SIDICs seemed to be a response to mechanical stress or injury.

**Figure 4:**
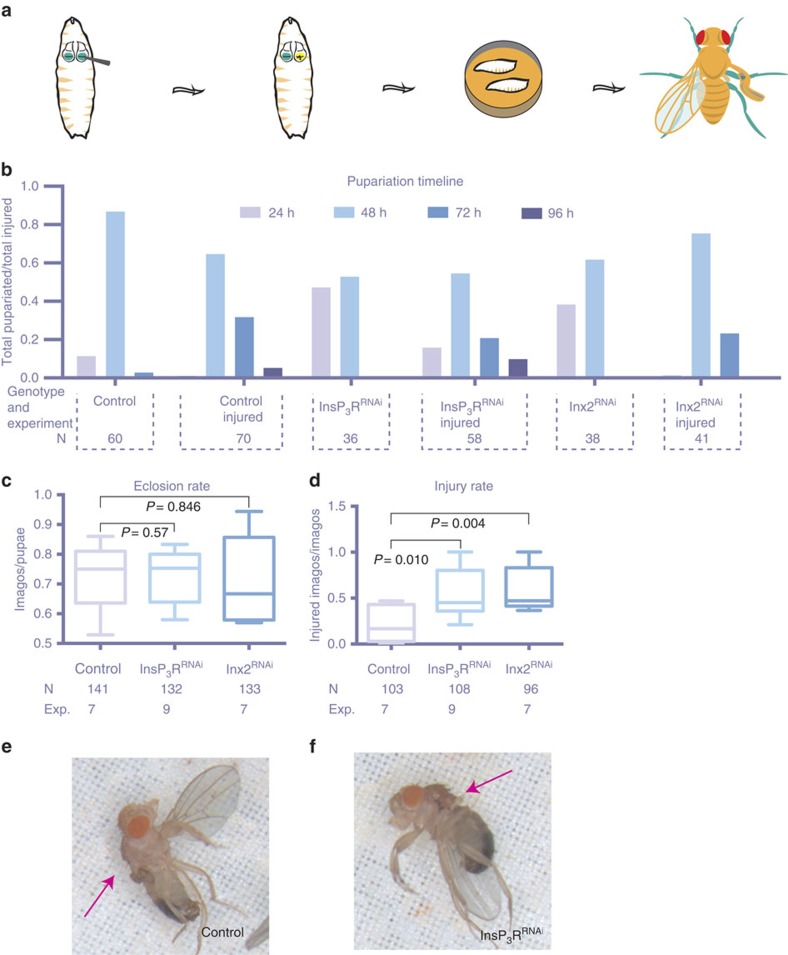
The SIDICs components support wound healing of *Drosophila* wing imaginal discs. (**a**) *In vivo* assay for recovery after mechanical injury. A blunt forceps tip is used to injure one disc per larva. The larvae that failed to heal eclose with heavily deformed or absent wings. (**b**) Pupariation timeline after mechanical injury. Control animals of all genotypes pupariate mainly during the first 48 h after a mock manipulation (no injury). Mechanical injury induced a delay in pupariation for all genotypes. Fly strains: *nub>GCamP6s X yw, nub>GCamP6s X UAS-InsP*_*3*_*R*^*RNAi*^ (NIG 1063-R1), *nub>GCamP6s X UAS-Inx2*^*RNAi*^ (BL 29306). (**c**) The eclosion rate after mechanical injury is not affected by RNAi lines against the key SIDICs components InsP_3_R and Inx2: yw (0.72±0.11, 7 experiments, *n*=103/141 larvae); InsP_3_R^RNAi^ (0.73±0.09, 8 experiments, *n*=108/132 larvae); Inx2^RNAi^ (0.71±0.15, 7 experiments, *n*=94/133 larvae). ‘Whiskers': min–max. *P*-values: Student's *t*-test. Fly strains: *nub>GCamP6s X yw, nub>GCamP6s X UAS-InsP*_*3*_*R*^*RNAi*^ (NIG 1063-R1 (6 exp.) or BL25937 (2 exp.)), *nub>GCamP6s X UAS-Inx2*^*RNAi*^ (BL 29306). (**d**) Average injury rate after mechanical injury, per round of experiment. InsP_3_R^RNAi^ and Inx2^RNAi^ lead to a clear increase in the amount of injured animals: yw (0.22±0.19, 7 experiments, 103 animals); InsP_3_R^RNAi^ (0.56±0.26, 9 experiments, 108 animals, *P*=0.010); Inx2^RNAi^ (0.57±0.2, 7 experiments, 96 animals *P*=0.004). ‘Whiskers': min–max. *P*-values: Student's *t*-test. Fly strains: *nub>GCamP6s X yw, nub>GCamP6s X UAS-InsP*_*3*_*R*^*RNAi*^ (NIG 1063-R1 (6 exp.)–BL25937 (2 exp.)), *nub>GCamP6s X UAS-Inx2*^*RNAi*^ (BL 29306). (**e**) Control animal that failed to heal after mechanical wounding. It is noteworthy that the internal control wing is normal and can be easily differentiated from the injured wing. This picture is a close up view from Supplementary Fig. 6. Fly strains: *nub>GCamp6s X yw.* (**f**) InsP_3_R^RNAi^ animal that failed to heal after mechanical wounding. It is noteworthy that the internal control wing is warped but can be easily differentiated from the injured wing. This picture is a close up view from Supplementary Fig. 6. Fly strains: *nub>GCamP6s X yw, nub>GCamP6s X UAS-InsP*_*3*_*R*^*RNAi*^ *(BL 25937).*

Therefore, we wondered whether the ability of wing discs to recover after a mechanically induced injury was impaired in RNAi backgrounds that abrogate SIDIC wave activity in the wing pouch. For this purpose, we developed an *in vivo* mechanical wounding assay for wing discs and scored for adults that eclosed with damaged wings. Here we damaged one imaginal wing disc—by punching it carefully through the cuticle under direct observation with a fluorescent compound microscope—and scored for imagos with deformed wings (Fig. 4a). It is noteworthy that both InsP_3_R^RNAi^ and Inx2^RNAi^ expression alone resulted in slightly deformed adult wings (Supplementary Fig. 5) (SERCA^RNAi^ results in heavily deformed adult wings; hence, we excluded this genotype from the analysis; Supplementary Fig. 5). To compensate for this, we only injured one wing disc, whereas the other provided an internal control. Crucially, the initial increase in reporter activity after injury can be used to visually control that the disc has indeed been injured, and that only one disc has been struck. Injured wings that had not healed properly resulted in heavily deformed stumps and could be unambiguously identified (Fig. 4e,f and Supplementary Fig 6). Importantly, both wounding and scoring were performed ‘blind' such that the experimenter ignored the genotypes of the animals being wounded/analysed.

*Drosophila* larvae delay their development after an injury, to allow the wounded organ to repair itself before pupariating^33^,^34^. Consistent with this, an analysis of the pupariation timeline following mechanical wounding revealed the expected delay in development relative to non-injured animals (Fig. 4b). We did not record a clear difference in this delay between InsP_3_R^RNAi^ or Inx2^RNAi^ and control animals. Further, the eclosion rate (number of flies/number of pupae) of control and knockdown groups was very similar (Fig. 4c), indicating that the degree of injury inflicted on each group had indeed been approximately equal.

Finally, we monitored the ratio of animals with a damaged wing (Fig. 4e,f). Evaluated over multiple rounds of experiments (seven to nine), the average ratio of injured animals per experiment was 0.216 (±0.185, *n*=103, 7 experiments) for control animals, 0.556 (±0263, *n*=108, 9 experiments, *P*=0.010) for InsP_3_R^RNAi^ and 0.573 (±0.196, *n*=96, 7 experiments, *P*=0.004) for Inx2^RNAi^ (Fig. 4d). These results indicate that in the absence of SIDIC activity, wound healing and regeneration can proceed; however, the rate at which they fail increases greatly.

The SIDICs are induced by mechanical stress *in vivo* and the knockdown of two components required for SIDIC formation and propagation affects the ability of the wing disc to heal after an injury. Based on these results, we infer that the SIDICs probably constitute a response to mechanical stress that contributes to the recovery of the wing imaginal disc after injury. However, we cannot exclude that InsP3R and Inx2 have cell-autonomous (non-SIDIC related) functions during wound healing, and that their knockdown could negatively synergize with mechanical injury to perturb wound healing.

## Discussion

In this study, we describe the occurrence of slow, long-range ICWs in imaginal discs (SIDICs), *in vivo* and *ex vivo*. We identify the InsP_3_R, SERCA and Inx2 as necessary components for SIDICs generation and propagation. Finally, we found that the SIDICs constitute a response to mechanical stress, probably supporting the wound healing and regeneration.

In comparison with previously reported calcium signals, such as the calcium flashes observed in the embryo^15^ or the calcium transients in the pupal notum^16^, the SIDICs are different in several aspects: the propagation speed, the duration of the phenomenon and the latency between the source of stress and the generation of the wave. The dissimilarities in the type of calcium signal observed in wing discs and the embryo and pupal notum could reflect inherent differences in the composition of the tissues studied. Alternatively, the SIDICs and the calcium flashes may be encoding different types of information that fulfill different purposes during wound healing and regeneration. In the embryo, the calcium flashes recruit hemocytes^15^, whereas in the pupal notum they coordinate cell contraction^16^. To further study the function of the SIDICs *in vivo*, a more sophisticated *in vivo* imaging setup will need to be developed. The *ex vivo* setup, however, proves to be a valuable substitute.

The wing disc has been an exquisite model for developmental genetics. Our work expands the utility of this model by revealing that it can be employed for the study of calcium signalling and ICWs. Wound healing and regeneration probably require a complex interplay between developmental pathways and the ability to coordinate cells during morphogenetic movements. Interestingly, calcium signalling has been proposed to be at the nexus of many signaling pathways^1^,^2^,^3^ and this nexus function could be essential for its role during regeneration. It will be interesting to see whether the SIDICs link, and perhaps help to orchestrate, different signalling pathways and morphogenetic movements during wound healing and regeneration of *Drosophila* imaginal wing discs.

## Methods

### *Ex vivo* imaging

*Ex vivo* imaging chambers were assembled with a life cell imaging dish (Zell-Kontakt) and a Millicell standing insert (PICMO1250) that we modified by removing its feet with a scalpel. Briefly, the wing discs were placed at the centre of the imaging disc, apical side facing down, in 20 μl of culture medium. Next, the modified insert was gently placed on top of the discs, thereby trapping the discs under the membrane. Finally, 200 μl of culture medium were added inside the insert. It is noteworthy that we did not employ an alginate gel in this study as previously described^17^. We used WM1 as culture medium: Schneider's medium (Sigma), 6.2 μg ml^−1^ bovine insulin (Sigma) and 5% Fly extract (home made)^17^. Time-lapse recording was performed on a Zeiss-Andor Spinning disc microscope equipped with an Ixon3 camera and a × 25 Zeiss Neofluor water immersion objective. GCamp6s was excited with a 488 laser. Imaging was performed in a dark room at 21 °C. We acquired three-dimensional time-lapses with the IQ2 software. *Z*-stacks were acquired every 10 s with 15% laser power, a gain of 50 and an exposure of 60 ms.

### *In vivo* imaging of squeezed larvae

Larvae were glued dorsally unto double-sided adhesive tape on a microscope slide. Imaging was performed with a Zeiss Axiovert microscope using a × 10 air objective under the control of ZEN (Zeiss). We used a Zeiss MRm camera with a 200 ms exposure and acquired images every 330 ms.

### *In vivo* imaging of immobilized larvae

Handmade imaging chambers were constructed as shown in Fig. 1. Larvae were glued ventrally on a microscope slide on double-sided adhesive tape. Two ‘chamber walls' consisting of three layers of sticky tape were deposited adjacent to the larvae. Finally, a coverslip was added on top of the chamber. This setup enables one to gently flatten the larvae to better position the wing discs perpendicular to the objective and provide a source of mechanical stimulation. Time-lapse imaging was performed on an Axioplan 2 microscope equipped with an Axiocam HRc under the control of Axiovision 4.7. Exposure was set to 100 ms and the time interval to 6 s.

### Staging

We staged larvae by restricting egg deposition to ∼8–12 h. Before conducting experiments, larvae were sorted visually under a binocular to refine the staging according to the following criteria: size, length, body colour and appearance of the spiracles.

### RNAi experiments

Crosses were kept at 25 °C and shifted to 29 °C, 24 h after egg deposition, to increase Gal4 and RNAi activity.

### *Inx2* ^*G0157*^ clone induction

*w, inx2*^*G0157*^*, FRT19A/FM7c*; were crossed to *ubi>mRFPNLS,w,hsflp,FRT19; nubbin-Gal4,GCamP6s; MKRS/Tm6b*, maintained at 25 °C and passed every 24 h. Clone induction was done 48 h after egg deposition at 37 °C for 30 min. *inx2*^*G0157*^*, FRT19A/ubi>mRFPNLS,w,hsFLP,FRT19*; *nubbin-Gal4,GCamp6s; +/Tm6b* roaming larvae were selected with a fluorescent binocular microscope.

All *Drosophila* strains used are listed in the Supplementary Methods.

### Statistics

All results were tested for normality (D'agostino and Pearson Omnibus normality test), to define which statistical test to perform. However, as normality can be difficult to judge in small sample sizes, we performed Student's *t*-tests and Mann–Whitney *U*-tests for all comparisons. All the results presented in this study were significant at a 95% threshold for both tests. We show *t*-tests in this study, as all our samples passed the normality test. All tests were performed with the software package PRISM from Graph Pad and Excel from Microsoft. Sample sizes were not determined before performing the experiments. No sample exclusion criteria were employed. For mechanical wounding assay the following blinding strategy was followed: genotypes were replaced by a numeric code unknown to the experimenter and changed for each experiment.

### Mechanical wounding assay

Four-day-old larvae of the correct genotype were collected with a sieve, rinsed with water and selected for GCamp6s basal fluorescence with a binocular microscope. Next, the larvae were dried with a paper towel and glued, ventrally, to a microscope slide covered with double-sided adhesive tape. A blunt forceps was then used to tap the wing discs without piercing the cuticle, while monitoring GCamP6s fluorescence to detect the injury. Afterwards, the larvae were detached from the adhesive tape with a drop of water and transferred to an apple agar petri dish. Recovering larvae were then placed in a fly incubator at 25 °C. At due time, freshly eclosed flies were anaesthetized with CO_2_ on a fly pad and scored for injured wings.

### Time-lapse processing

First, the original files were transformed into three-dimensional time lapses with the stack to hyperstack function in Fiji (http://fiji.sc/Fiji). Second, the hyperstacks were subject to a maximum intensity projection to generate two-dimensional time lapses. All calculations were performed on these two-dimensional time lapses.

### Cellular calcium pulse duration calculation

For each sample, we selected a region of interest (ROI) of approximately the size of a cell, randomly, in the trajectory of a wave. We used the ROI Intensity Evolution plugin of ICY (http://icy.bioimageanalysis.org/)^35^ to produce an excel file of the average fluorescence intensity per time frame of the ROI. Finally, we used Excel to calculate the duration of an average cell pulse.

### Wave magnitude calculation

The area of the pouch was measured in ICY^35^ by employing the baseline fluorescence of GCamP6s and morphological landmarks. The magnitude of a wave was calculated in the following way. First, the time lapse was cropped in time to include only one wave or 10 min. The 10-min crops were required for genotypes in which wave activity was diminished. Finally, the area covered by the wave was calculated by performing a maximum intensity projection over time of the 10-min per one wave time lapses with the Intensity Projection plugin in ICY^35^. The wave magnitude was calculated as wave area per pouch area.

### Wavefront speed calculation

We calculated the wavefront speed manually by defining two semi-parallel lines in (superimposed on the time lapses and perpendicular to the wavefront) in ICY^35^ and measuring the time required by the wavefront to cross the distance between the two lines.

### Wave period calculation

For each time lapse, we generated a ROI outlining the pouch by employing the baseline fluorescence of GCamP6s and morphological landmarks. Next, we used the ROI Intensity Evolution plugin of ICY^35^ to produce an Excel file of the average fluorescence intensity of the ROI per time frame. Finally, we used Excel to calculate the average interval in between peaks of fluorescence for the ROI.

### Data availability

The authors declare that all data supporting the findings of this study are available within the article and its Supplementary Information files or from the corresponding author upon reasonable request.

## Additional information

**How to cite this article**: Restrepo, S. & Basler K. *Drosophila* wing imaginal discs respond to mechanical injury via slow InsP_3_R-mediated intercellular calcium waves. *Nat. Commun.* 7:12450 doi: 10.1038/ncomms12450 (2016).

## Supplementary Material

Supplementary Information Supplementary Figures 1-6 and Supplementary Methods

Supplementary Movie 1 A slow imaginal disc intercellular calcium wave observed under *ex vivo* conditions. Note the recurring nature of the SIDICs *ex vivo*.

Supplementary Movie 2 A slow imaginal disc intercellular calcium wave observed under *in vivo* conditions after mechanical injury.

Supplementary Movie 3 A slow imaginal disc intercellular calcium wave observed under *in vivo* conditions after mechanical injury that recurs.

Supplementary Movie 4 *Ex vivo* time-lapse showing that InsP3R knockdown prevents SIDIC formation.

Supplementary Movie 5 *Ex vivo* time-lapse showing that SERCA knockdown prevents SIDIC formation.

Supplementary Movie 6 *Ex vivo* time-lapse showing that Innexin2 knockdown prevents SIDIC formation.

Supplementary Movie 7 *Ex vivo* time-lapse showing that the SIDICs cannot readily propagate across *inx2* mutant clones.
