# Supplementary material for: Drosophila wing imaginal discs respond to mechanical injury via slow InsP3R-mediated intercellular calcium waves
Source: Nat Commun. 2016 Aug 9;7:12450. doi: 10.1038/ncomms12450 (PMC4980486; doi:10.1038/ncomms12450)
Supplement: Supplementary Information — Supplementary Figures 1-6 and Supplementary Methods [file ncomms12450-s1.pdf]

Supplementary Figures

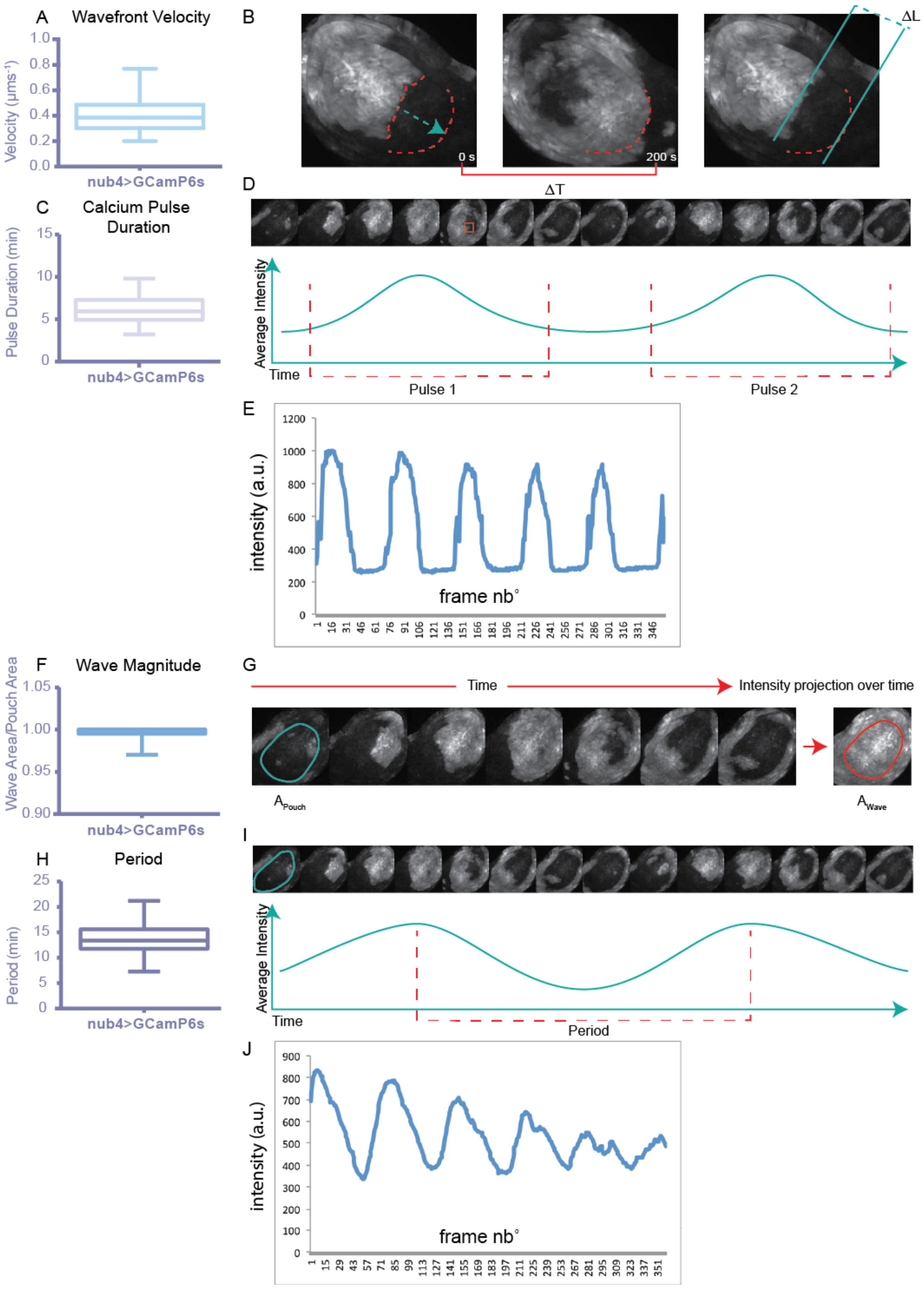

Supplementary Figure 1

A. The average wavefront velocity is of 0.41 μms^{-1} +/- 0.17 (n=10). A'. Illustration of the calculation method for the wavefront velocity. "Whiskers": min-max. *nub>GCamp6s X yw.*

B. Schematic representation of how the wavefront velocity was calculated. *nub>GCamp6s X yw.*

C. During a wave, the peak of intracellular calcium mobilization per cell is on average of 6.18 min +/- 1.97 (n=10). "Whiskers": min-max. *nub>GCamp6s X yw.*
D. Illustration of the calculation method for the calcium pulse duration. *nub>GCamp6s X yw.*
E. Representative graph of cell pulse duration. Y-axis, mean GCamp6s fluorescence intensity in a one cell sized ROI. X-axis, frame no. (10 sec. intervals). *nub>GCamp6s X yw.*
F. On average during each SIDIC 0.99 +/- 0.01 (n=10) of the pouch are recruited. E' Illustration of the calculation method for the wave magnitude. "Whiskers": min-max. *nub>GCamp6s X yw.*
G. Illustration of the calculation method for the wave magnitude. *nub>GCamp6s X yw.*
H. *Ex vivo*, the SIDICs recur with an average period of 13.9 min +/- 3.4 (n=22). *nub>GCamp6s X yw.*
I. Illustration of the calculation method for the wave period. "Whiskers": min-max. *nub>GCamp6s X yw.*
J. Representative graph of wave period duration. Y-axis, mean GCamp6s fluorescence intensity in a whole pouch size ROI. X-axis, frame no. (10 sec. intervals). *nub>GCamP6s X yw.*

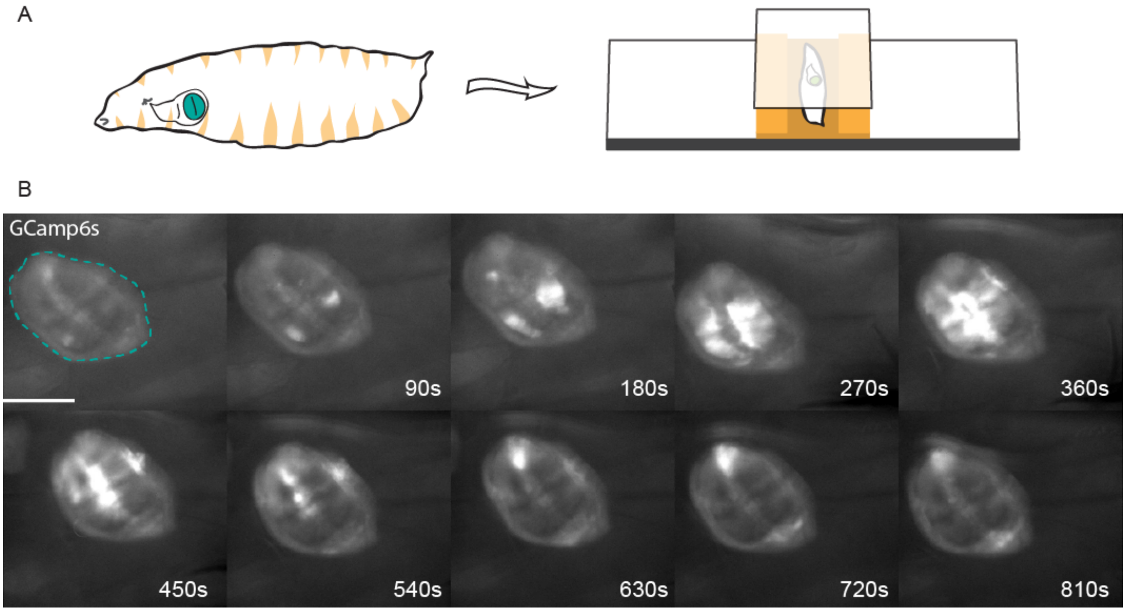

Supplementary Figure 2

A. *In vivo* imaging setup for squeezed larvae. Animals were glued to a microscope slide, ventrally, with double sided adhesive tape. Additional layers of tape were used to construct a chamber surrounding the larva. Finally the larva was squeezed with a microscope coverslip. We employed an upright widefield fluorescence microscope.

B. SIDICs could also be observed with this setup. However the wave were generally visible only shortly after sample mounting, indicating that the mounting procedure and the squeezing might have triggered the SIDICs. Scale bar: 100 µm. *nub>GCamp6s X yw.*

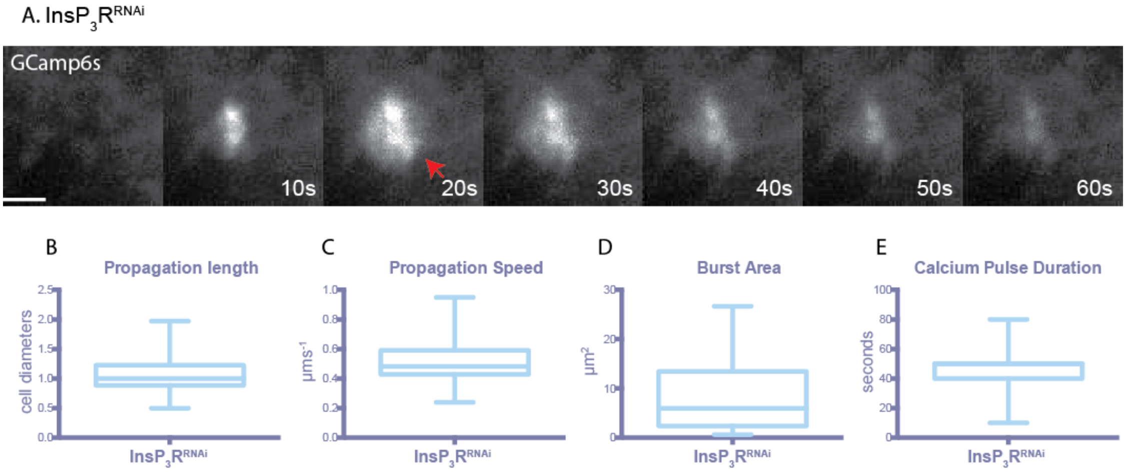

Supplementary Figure 3

A. Close-up view of a calcium burst during an $InsP_3R^{RNAi}$ time-lapse (25X magnification and digital crop). Arrowhead, burst propagation. Scale bar: 10 µm. *nub>GCamP6s X UAS-InsP₃R*^{RNAi} (NIG 1063-R1).
B. Propagation length of a calcium burst (cell diameters), n=10. "Whiskers": min-max. *nub>GCamP6s X UAS-*$InsP_3R^{RNAi}$ (NIG 1063-R1).
C. Propagation speed of a calcium burst (μms^{-1}) n=10. "Whiskers": min-max. *nub>GCamP6s X UAS-*$InsP_3R^{RNAi}$ (NIG 1063-R1).
D. Burst area of a calcium burst (μm^2) n=10. "Whiskers": min-max. *nub>GCamP6s X UAS-*$InsP_3R^{RNAi}$ (NIG 1063-R1).
E. Calcium burst duration (sec.) n=10. "Whiskers": min-max. *nub>GCamP6s X UAS-*$InsP_3R^{RNAi}$ (NIG 1063-R1).

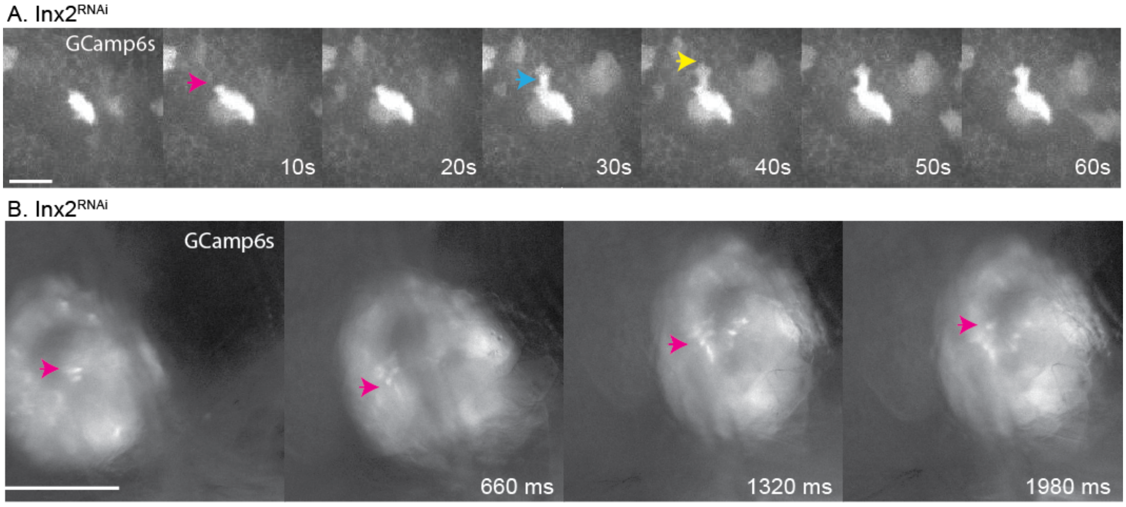

Supplementary Figure 4

A. Close-up view of an Inx2^{RNAi} time-lapse (63X magnification and digital crop). Note that sometimes short-range calcium transients can propagate for 1-3 cell diameters. Arrowheads, new cells in a short-range calcium transient. Cell junctions are marked with DECADHERIN::GFP. Scale bar: 10 µm. *decadherin::GFP; UAS-Inx2^{RNAi} (BL 29306) X c765-Gal4/Tm6b*

B. Similar calcium "sparkles" could be observed *in vivo* but only after mechanical stimulation, indicating that they constitute a response to stress/injury rather than a constitutive effect on gap junction knockdown. Arrowheads, calcium "sparkles". Scale bar: 100 µm. *nub>GCamP6s X UAS-Inx2^{RNAi}* (BL 29306).

A.

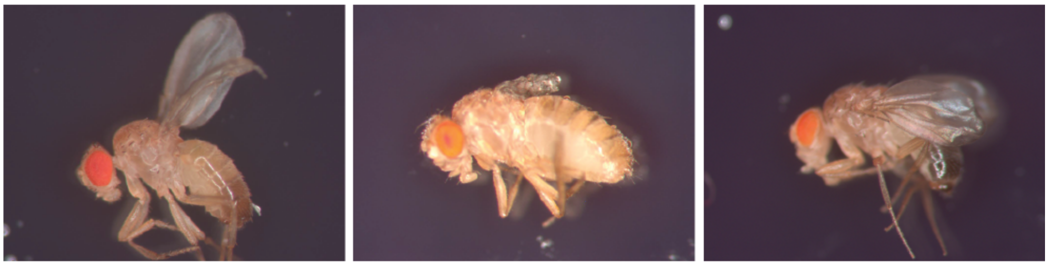

Supplementary Figure 5

A. Adult phenotypes of $InsP_3R^{RNAi}$ (NIG 1063R-2), $SERCA^{RNAi}$ (BL25928) and $Inx2^{RNAi}$ (BL 29306). Here we show a stronger RNAi line for $InsP_3R$ than used in the regeneration experiments because the phenotypes of BL25937 and NIG 1063R-1 are subtler and harder to photograph clearly. *nub>GCamP6s X UAS-*$InsP_3R^{RNAi}$ (NIG 1063R-2) or *UAS-*$Inx2^{RNAi}$ (BL 29306) or *UAS-*$SERCA^{RNAi}$ (BL 25928).

A. nub4>GCamP6s X yw

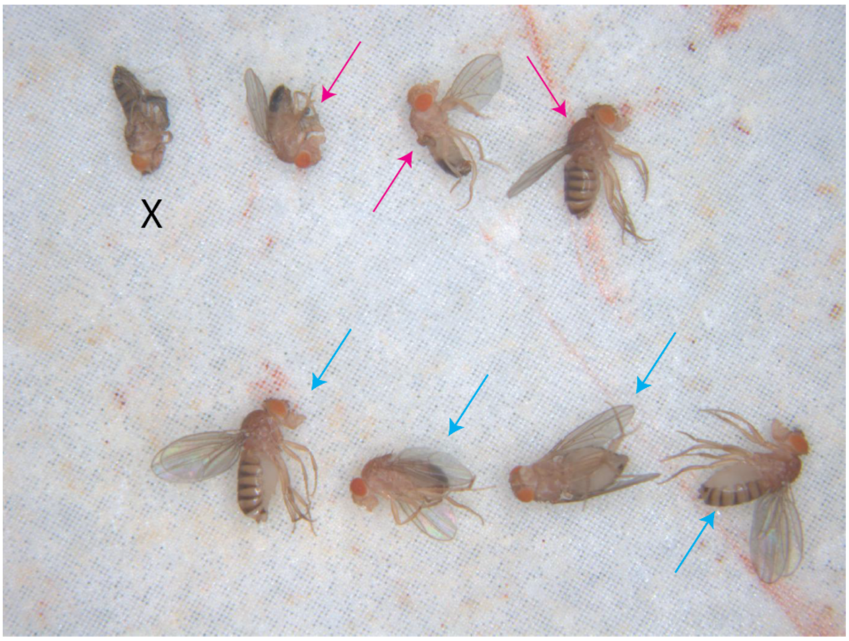

B. nub4>GCamP6s X InsP$_3$R^{RNAi}

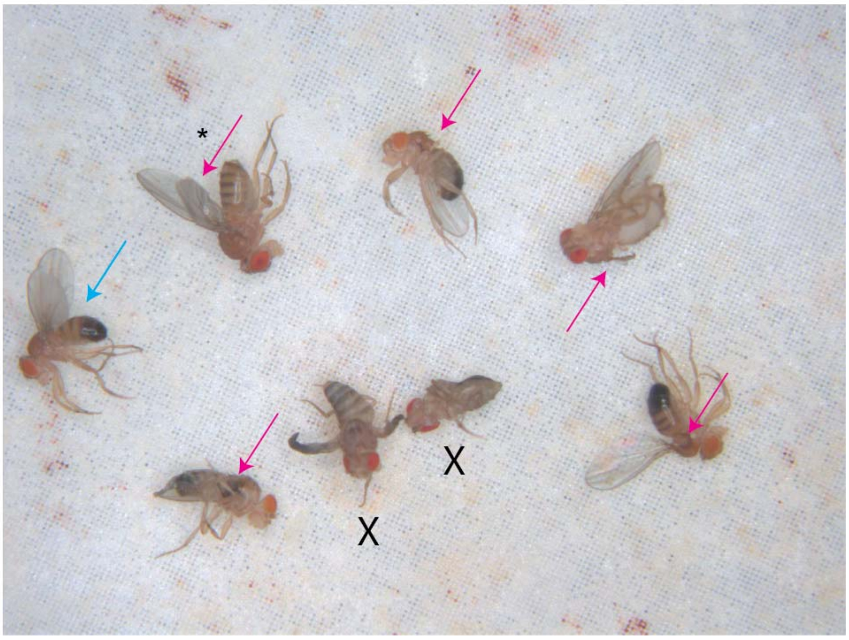

Supplementary Figure 6

A. Control experiment. Note three animals with a clear injury (magenta arrows) and four intact (cyan arrows) and one dead imago (black X). Dead imagos were considered ambiguous and not scored as injured. Not all animals from this experiment are shown here for clarity (the injury ratio shown here is not representative). *nub>GCamP6s X yw.*

B. InsP$_3$R^{RNAi} experiment. Here we show five animals with a clear injury (a weaker phenotype is highlighted with a black asterisk). The animals with ambiguous phenotypes were not scored (black X). *nub>GCamP6s X UAS-InsP$_3$R^{RNAi}* (BL 25937).

Supplementary Methods

Fly lines

1. *y,w, hs>flp; nubbin-Gal4, GCamP6s/CyO; MKRS/Tm6b*
2. *y,w,hs>flp; nubbin-Gal4; MKRS/Tm6b was recombined with yw,hsflp; P{20XUAS-IVS-GCaMP6s}attP40/ CyO; MKRS/Tm6b*
3. *w[1118]; P{y[+t7.7] w[+mC]=20XUAS-IVS-GCaMP6f} attP40* Bloomington Drosophila Stock Center number 42746
4. *y,w*
5. *y,w, hs>flp ;;c765-Gal4/Tm6b*
6. *y,w, hs>flp; decadherin::GFP; BL 29306/Tm6b*
7. *ubi>mRFP.nls,w,hs>flp,FRT19; nubbin-Gal4,GCamP6s; MKRS/Tm6b*
8. w^{67c23} *P{lacW}Inx2*^{G0157} *P{neoFRT}19A/FM7c; P{ey-FLP.N}5* Drosophila Genetic Resource Center number 111854
9. *insP3R*^{ug3} described in Joshi et al., 2004 [26]. Kindly provided by Dr. Gaiti Hasan
10. *insP3R*^{wc361} described in Joshi et al., 2004 [26]. Kindly provided by Dr. Gaiti Hasan

RNAi lines

Abbreviations used for origin:

Bloomington Drosophila Stock Center, BL

National Institute of Genetics, NIG

**Target**	**CG**	**RNAI lines**
SERCA	CG3725	**BL 25928, BL 44581**
$InsP_3R$	CG1063	**BL 25937, NIG1063R-1, NIG1063R-2**
Innexin2	CG4590	**BL 29306**, **UAS-wizInx2** (Described in Lechner et al., 2007 [28]. Kindly provided by Dr. Michael Hoch.)
